# Vesicular Stomatitis Virus Transmission: A Comparison of Incriminated Vectors

**DOI:** 10.3390/insects9040190

**Published:** 2018-12-11

**Authors:** Paula Rozo-Lopez, Barbara S. Drolet, Berlin Londoño-Renteria

**Affiliations:** 1 Department of Entomology, Kansas State University, Manhattan, KS 66506, USA; paularozo@ksu.edu; 2 United States Department of Agriculture, Agricultural Research Service, Arthropod-Borne Animal Diseases Research Unit, Manhattan, KS 66502, USA

**Keywords:** vesicular stomatitis virus, vector, transmission, mosquito, sand fly, black fly, *Culicoides* midges

## Abstract

Vesicular stomatitis (VS) is a viral disease of veterinary importance, enzootic in tropical and subtropical regions of the Americas. In the U.S., VS produces devastating economic losses, particularly in the southwestern states where the outbreaks display an occurrence pattern of 10-year intervals. To date, the mechanisms of the geographic spread and maintenance cycles during epizootics remain unclear. This is due, in part, to the fact that VS epidemiology has a complex of variables to consider, including a broad range of vertebrate hosts, multiple routes of transmission, and an extensive diversity of suspected vector species acting as both mechanical and biological vectors. Infection and viral progression within vector species are highly influenced by virus serotype, as well as environmental factors, including temperature and seasonality; however, the mechanisms of viral transmission, including non-conventional pathways, are yet to be fully studied. Here, we review VS epidemiology and transmission mechanisms, with comparisons of transmission evidence for the four most incriminated hematophagous dipteran taxa: *Aedes* mosquitoes, *Lutzomyia* sand flies, *Simulium* black flies, and *Culicoides* biting midges.

## 1. Introduction

Vesicular stomatitis (VS) is an economically significant viral disease of cattle, horses, and swine, resulting in vesicular lesions of the gums, tongue, naso-oral mucosa, teats, and coronary bands [1]. Infection with vesicular stomatitis virus (VSV) does not typically result in high mortality rates; however, negative economic impacts to livestock producers can be devastating due to animal production losses and restrictions on animal movement to control the spread of disease [2,3,4]. In the United States, the economic impact of an outbreak of VS has been estimated at $100 to $200 per cow and a mean loss of $15,565 per ranch infected with VSV [5,6]. Additionally, VS causes significant alarm due to it being clinically indistinguishable from foot and mouth disease, a devastating viral infection of livestock eradicated from the U.S. in 1929 [1,7]. VSV is maintained in stable ecologic niches in Central and South America, where annual outbreaks infect a large percentage of susceptible species [1]. In the U.S., outbreaks with viral strains originating from these southern endemic regions occur primarily in southwestern states at approximately 10-year intervals [2,3,8]. Epidemiological factors which may contribute to this sporadic northern migration of specific endemic viral strains from Mexico into the U.S. are not clearly understood [8].

Information regarding transmission, duration, and geographic spread during epizootics is based largely on case reporting by veterinarians and limited entomological collections conducted during these sporadic outbreaks [4,9]. In addition to direct contact, aerosol, and fomites [1,10], virus transmission routes clearly involve insect vectors, such as mosquitoes [11], sand flies [12], black flies [13,14], and biting midges [15,16]. In terms of virus persistence in nature, serological surveys have shown that in addition to domestic livestock, many species of wild animals develop neutralizing antibodies to the virus [17,18,19,20]; however, a definitive natural host reservoir remains unclear and transmission cycles between vectors and wildlife have not been established [2]. This manuscript reviews relevant literature of VS epidemiology and VSV transmission, with an emphasis on the main species of proposed hematophagous dipteran vectors, and brings into focus components and mechanisms of transmission that are still understudied.

## 2. Etiological Agent

Vesicular stomatitis viruses are members of the family, Rhabdoviridae, genus, *Vesiculovirus* [21]. The virions are bullet-shaped and are generally 180 nm long and 65 nm wide [21]. The virion envelope, derived from the host cell, is arranged by an external phospholipid bilayer membrane that surrounds a very stable internal ribonucleoprotein core [22]. The genome of VSV consists of a negative sense single-stranded RNA, 11,161 nucleotides in length, encoding five major proteins: N (nucleocapsid or ribonucleoprotein), P (phosphoprotein), M (matrix protein), G (glycoprotein), and L (large protein or polymerase) [23,24,25]. The G transmembrane glycoprotein forms spikes on the envelope and mediates cellular recognition and fusion, allowing the viral entry and exit from the cell [26,27]. The M protein is located between the envelope and the nucleocapsid core, and participates in viral assembly and particle budding [28]. The nucleocapsid core is composed of the viral genome tightly wound around the viral N protein, forming an RNase-resistant core environment [29]. The P and L proteins combine to catalyze RNA-dependent RNA polymerization (RdRp) of genomic RNA and transcription of the mRNAs [23,29,30] in the sequential order of N-P-M-G-L [23,31,32]. For genomic replication, the RdRp initiates at a different 3’ end site and synthesizes a full-length positive-sense copy of the genome as a replication template [30]. Due to the low fidelity rate of RdRp, VSV has a high error rate for RNA transcription, which leads to great genetic diversity and quasispecies populations [2,33,34]. Transcription and replication mechanisms of VSV are complex and not fully understood, with laboratory and natural populations yielding variable information on genetic adaptability and maintenance. Under laboratory conditions, VSV shows great capacity for genetic change and rapid adaptation [2,35,36,37]. Conversely, in field conditions, VSV remains relatively stable, with evolutionary patterns defined by similar ecological conditions rather than geographical origin or immunological selection [2,8]. Genetic fitness studies designed to investigate evolutionary pressure on the genome of VSV alternating between insect and mammalian cellular environments suggest that the stability of field populations is not due to the need of the virus to constrain adaptation between host cell types [38].

VS viruses are classified by serotypes, which are similar in size and morphology, but generate distinct neutralizing antibodies in infected animals [39,40]. There are two distinct serotypes of VSV: Indiana (VSV-IN) and New Jersey (VSV-NJ), with the latter causing the majority of outbreaks in the U.S. [2,7,41,42]. Serotypes involved with the disease in livestock include VSV-NJ and VSV-IN types 1, 2, and 3 [7]. Serotypes, VSV-NJ and VSV-IN1, occur in North, Central, and parts of South America [2,8]. Serotype VSV-IN3 (or Alagoas) occurs in Brazil, and VSV-IN2 (or Cocal) occurs in Brazil and Argentina [35,43]. Other vesiculoviruses (Piry, Chandipura, Jurona, Carajas, Maraba, Calchaqui, Yug Bogdanovac, Isfahan, Perinet, and Porton-S) have been isolated from arthropods and mammals; however, these have not been shown to cause natural outbreaks in livestock [1,12,44,45].

## 3. Pathogenesis and Epidemiology

VSV infection occurs primarily in domesticated cattle, horses, swine, and rarely in llamas and humans [46]. Infection of horses, as reviewed by McCluskey and Mumford, is particularly significant in the U.S. [47]. The infection is typically short-lived and self-limiting, but secondary bacterial infections, such as mastitis, can occur, especially in dairy cattle and nursing mares [47,48]. Although the incubation period is variable, clinical disease usually develops after a period of 2 to 7 days [47]. Viral transcription and replication take place within just a few hours post-infection and the peak of viral replication ranges from 24 to 48 h post-inoculation [49]. A viremic phase with detectable infectious virus has not been shown in livestock [50], possibly due to the inhibitory effect of serum proteins, such as complement [51]; however, VSV RNA persists for months in a variety of tissues, such as lymphoid tissue [1,52].

Histologically, VSV infection can result in vesiculation, epithelial cell lysis, and severe interstitial edema, which appears with the infiltration of inflammatory cells [53]. VSV clinical pathology includes vesicle development in the mucosa and subsequent ruptures, leading to cavities filled with cellular exudates [54,55]. The route of VSV exposure influences the host responses and subsequent clinical disease [1,56,57,58] with vesicular lesions developing only at specific sites of inoculation, such as oral mucosa, the snout of pigs, teats of cattle, and coronary bands of pigs, cattle, and horses [47,48,53,58]. Lesions are considered extremely important for direct contact and vector-borne transmission due to high titers of virus in vesicular fluids, at the margins of damaged tissues, and in the copious amounts of saliva due to oral lesions [42,59].

VS is enzootic in tropical and subtropical regions of the Americas; however, it has been known to spread north into temperate zones in the U.S. and Canada during the summer months, appearing first in states along the Gulf of Mexico in April or May and later as far north as Manitoba, Canada [8,60]. In Central and South America, VS outbreaks are variable and often associated with the transitions between rainy and dry seasons [7]. In tropical and subtropical zones, VSV transmission is most common at the end of the rainy season or early in the dry season [35]. In temperate regions, VS outbreaks occur seasonally during summer, with the spread often following rivers, windward directions, and distinct ecological features, and usually end after the first hard frost [7,46,61,62].

In the U.S., VSV has been reported most frequently in the southwest and southeastern states; however, both regions show different patterns of occurrence [2]. In the southeast, yearly occurrences were reported until the mid-1970s, while in the southwestern states, VS outbreaks continue to occur sporadically in roughly 10-year intervals [2,8], with the most recent being the 2014–2015 outbreak [63]. Occasionally, outbreak viruses overwinter in a yet to be identified natural reservoir, the same viral genotype causing disease a second year, such as occurred during the 2004–2005 and 2014–2015 outbreaks [2,63,64]. In the second year, the spread is often further from flowing water, into dryer regions, suggesting a possible change in the primary vector species. Phylogenetic analyses of VSV lineages from the southwestern and southeastern outbreaks have shown distinct lineages for each region, with a common origin from endemic areas of Mexico [8]. In the southwestern region, the serotypes involved in outbreaks are VSV-NJ and VSV-IN1, and in the southeastern region, VSV-NJ [2,8]. The New Jersey serotype is responsible for the majority (80%) of the outbreaks in the U.S., and Indiana 1 for the remainder [48].

## 4. Vectors and Mechanisms of Transmission

During outbreaks, VSV spreads quickly within animal herds by direct contact and fomites [1,10]. Infected animals salivate excessively and release between 4 and 6 logs of virus per milliliter of saliva [59]. Virus-laden saliva and vesicular exudates easily contaminate facilities and the environment, allowing an efficient animal to animal or fomite to animal transmission [3,42,59,65]. Research has shown that insects also play a significant role in VSV dissemination in two proposed ways, as mechanical vectors by either biting or nonbiting flies, or as biological vectors by hematophagous biting flies [60,66,67,68,69]. Currently, several aspects of VSV transmission are not well understood, particularly: (1) Where and how the proposed insect vectors acquire the virus in nature; (2) if the virus isolated from insects captured in field collections during epizootics correspond to biological or mechanical transmission and are epidemiologically relevant; (3) if any of the currently incriminated or suspected insect species are involved in VSV maintenance during inter-epidemic periods; and (4) if the low or often undetectable viremia in infected mammals indicates that other transmission mechanisms may be involved for vectors to become infected.

### 4.1. Mechanical Vector Transmission

In addition to fomites, mechanical transmission occurs with insects and is typically characterized by physical viral transport with little insect specificity and by the absence of an incubation period [68]. In the case of VSV infection, domestic animals have low, transient viremia during infection [1], which complicates the transmission cycle dogma of a vector-borne disease. However, vesicular lesions contain high virus titers and are clearly important for animal to animal direct contact transmission [42,59]. Consequently, high viral titers in the skin associated with vesicular lesions may be a source of the virus for vector mechanical transmission [3,66]. In the case of hematophagous pool-feeding flies, such as sand flies, black flies, and *Culicoides* midges, VSV can be acquired by feeding on or near vesicular lesions or on the intact skin contaminated by virus-laden saliva [49,56,70].

The plausibility for mechanical transmission of VSV is furthered by the large number of species found to carry the virus under natural conditions. During epizootics, VSV has been isolated from non-hematophagous insects, such as houseflies (Muscidae), eye gnats (Chloropidae), and Anthomyiidae flies [60,67]. Furthermore, the ability of grasshoppers to become infected and replicate VSV to high titers after ingesting virus-contaminated pasture plants has been proposed as a possible explanation for the long-distance spread and outbreaks in pastured herds far from water sources [71,72]. Laboratory experiments have shown that fly species, including horseflies (*Tabanus*), deer flies (*Chrysops*), mosquitoes (*Aedes* and *Culex*), and stable flies (*Stomoxys*), captured in large numbers in livestock stables were capable of transmitting VSV to susceptible embryonated eggs, and that cows exposed to bites of infected flies developed neutralizing antibodies to VSV [68].

### 4.2. Biological Vector Transmission

Biological transmission is characterized by a high degree of vector specificity and an extrinsic incubation period (EIP) during which virus proliferation occurs before it reaches transmission-related organs (salivary glands, eggs). Despite the lack of a demonstrable viremia in infected animals, several aspects of outbreaks suggest that VSV is vector-borne by hematophagous insects. Seasonal occurrence of VS in temperate regions peaks during summer and fall, which corresponds with peak vector populations, and reported cases typically stop after the first hard freeze [7,46,59]. There is also a tendency for outbreaks to recur in certain geographical areas, particularly near running or standing water, which are prime habitats for black flies and *Culicoides* midges, respectively [73]. Finally, the rapidity and manner of spread, in the absence of animal movement, suggests transportation of the virus by flying insects [59,73]. To date, only sand flies have been experimentally shown to transovarially transmit VSV to their offspring [17,74]. As with most arbovirus transmission cycles, vertebrate hosts typically act as amplifying reservoirs capable of producing sustained levels of viremia. Serological surveys have shown that species, such as bats, deer, and monkeys, living in endemic areas develop neutralizing antibodies against the virus [2,18,20], and that small grass-eating rodents, such as cotton rats [18,19] and deer mice [75,76], might play a role in viral maintenance. However, a definitive natural viremic host reservoir for VSV is yet to be identified [2].

The undetectable viremia of VSV in vertebrate hosts suggests that biological vector species need to be exceptionally susceptible to acquire infection from blood feeding [77]. However, there is no sufficient data showing that low viremias cannot infect vectors. Vector species may still become infected depending on the number of hosts with this viremia, the duration of the viremia, the number of virions required to initiate a replicating infection in a vector, and the number of vectors feeding on these hosts [78]. To date, two alternatives have been proposed other than blood from viremic hosts as sources for virus uptake by feeding vectors: Infection by co-feeding with infected insects [66,79], and probing or feeding on or near epidermal vesicular lesions of clinically infected animals [60]. The mouthparts of pool-feeders, which cut the epidermis and ingest blood that pools into the wound (sand flies, black flies, and *Culicoides* midges), inherently encounter more skin microbes than capillary vessel-feeders, which insert a proboscis directly into a blood vessel (mosquitoes). Thus, pool-feeders are likely to ingest more virus on contaminated skin surfaces and may, therefore, play a more important role in transmitting epidermal lesion-sourced VSV than mosquitoes. While the complex natural transmission cycle of VSV is still not completely elucidated, many laboratory studies have demonstrated the ability of specific hematophagous dipterans to transmit VSV, and epidemiological studies have associated clinical VS cases in cattle, horses, and swine with exposure to four main species: Mosquitoes, sand flies, black flies, and *Culicoides* biting midges [14,67,80,81,82].

#### 4.2.1. Mosquitoes (Diptera: Culicidae)

*Aedes* mosquito populations were found infected during epizootics of VSV in Mexico [77], which suggested that Culicidae might play a role in transmission. However, the low number of virus isolations in comparison with the high number of mosquitoes tested provided little confirmation on whether mosquitoes act as mechanical or biological vectors [77]. In laboratory experiments, VSV multiplication in *Aedes aegypti* was observed when the mosquitoes were infected by intrathoracic injection and by feeding mosquitoes on sugar solutions containing virus [83]. Additionally, analysis of infectious virus particles revealed that *Ae. aegypti* tissues supported the growth of both VSV serotypes after intrathoracic injection [11,80]. The multiplication of Indiana and New Jersey strains showed slightly similar infection patterns reaching the highest viremias between 2 and 3 days after injection and lower peaks after 8 days when the infection was reduced in all organs, but remained high in the salivary glands only for VSV-IN [11,80]. Interestingly, higher doses of virus inoculation caused no increase in mosquito whole body virus titers [80].

Previous research also has shown that *Ae. aegypti* mosquitoes are capable of transmitting VSV-IN and VSV-NJ to baby mice at rates of 11% and 5%, respectively, following intrathoracic inoculation [80]. *Ae. triseriatus* mosquitoes, intrathoracically inoculated with VSV-NJ, have also shown to be capable of virus delivery and potentiation of infection in mice [84]. Limesand et al. (2000) showed that *Aedes* infected mosquitoes exhibited altered behaviors, such as taking longer periods of time to reach engorgement and probing more times than uninfected mosquitoes [84]. The mosquito probing led to mouse seroconversion, demonstrating that VSV could be potentially transmitted without the need for engorgement [84]. Although *Aedes* mosquitoes have been shown to exhibit some vectorial capacity for VSV transmission under these laboratory conditions, intrathoracic injections bypass infection and escape barriers of the midgut and therefore does not prove biological vector competence. Thus, *Aedes* spp. mosquitoes remain controversial as a competent vector species for VSV in nature.

#### 4.2.2. Sand Flies (Diptera: Psychodidae)

Sand flies were one of the earliest insect species found infected with VSV in natural habitats [17] and historically have been the most frequent insects associated with natural infection [12,17]. In the U.S., VSV-NJ was enzootic in the feral pig population on Ossabaw Island in Georgia until the early 2000s, where multiple entomological collections isolated this serotype from *Lutzomyia shannoni* [85,86,87]. In tropical areas where VSV-IN is endemic, large numbers of sand flies captured during Leishmaniasis surveillance have also contributed to the knowledge of VSV infections from natural habitats [12]. Furthermore, laboratory evidence of virus multiplication, bite transmission, and by experimentally infected sand flies suggests these pool-feeding insects as capable vectors of VSV [81,88,89,90]. For oral infection with VSV-IN, the EIP for *Lutzomyia trapidoi* is reported to be 3 days [81] and 5 to 6 days for oral infection of *L. shannoni* with VSV-NJ [89].

Among all the hematophagous insects implicated in VSV transmission, only sand flies have been shown to be competent of transovarial transmission (TOT) [88,90]. This type of generation to generation transmission suggests a mechanism for maintenance of VSV in nature without a vertebrate host. TOT experiments have shown VSV replication during the development of sand flies and F1 virus titers comparable to those found in wild infected sand flies, with 1.1% of the F1 progeny infected by VSV-NJ and 20% of the F1 progeny infected by VSV-IN [88]. Additionally, infected F1 adult females were able to transmit the virus through bites into susceptible hamsters [88]. Furthermore, VSV was detected in F2 larvae, suggesting that the virus can be passed from more than one generation [88].

Sand flies appear to be important vectors in endemic areas of Central and South America. However, several factors lead to open questions about their capacity for VSV transmission and their contribution with the outbreaks in the central and western regions of the U.S. First, *Lutzomyia* species often have very specific habitat requirements and are only found in regions with suitable climates, habitat types, and host animals [91]. Second, sand flies have a limited flight range and patterns of sporadic activity [91], consequently, they are not thought to spread the virus long distances. Lastly, the TOT rates of 20% for VSV-IN and 1.1% for VSV-NJ would not sustain the virus indefinitely in the absence of infected adults, venereal transmission by infected males, or high viremia reservoirs to replenish the virus in fly populations under natural conditions. The disappearance of VSV on sandy fly-infested Ossabaw Island with the eradication of native feral swine is a good case in point [92].

#### 4.2.3. Black Flies (Diptera: Simuliidae)

Black flies are the most well-characterized, biologically competent vectors of VSV. Several studies have detected VSV in wild simuliid populations during epizootics [3,60,67]. During the 1982 outbreak in Colorado, VSV-NJ was isolated from black flies with such high titers that virus replication was likely [13,60]. Biological transmission was first suggested when VSV-NJ intrathoracically inoculated black flies were able to elicit neutralizing antibody in mice [74]. Following experiments showed clear biological transmission of VSV by black flies to domestic swine [93] and domestic cattle [56].

Wild populations of black flies of the genus, *Simulium*, that were fed Mexican and Western U.S. isolates of VSV-NJ supported viral replication for at least 10 days; however, not all species were capable of secreting virus in their saliva [94]. *Simulium bivittatum* and *S. longithallum* showed virus replication, but not dissemination into the salivary glands. Only *S. notatum* was found to be a competent laboratory biological vector for VSV-NJ with virus detected in the saliva of infected flies [94,95]. In the case of vector competence of wild-caught black flies for VSV-IN, *S. vittatum* and *S. notatum* showed virus in the saliva following oral infection, indicating that they are competent laboratory vectors [9]. Immunolocalization of VSV-NJ in *S. vittatum* via feeding of virus or intrathoracic injection showed initial infection of the gut followed by subsequent spread into the salivary gland [96]. These processes seem to be blocked in older flies, decreasing their vectorial capacity, therefore, only younger females are competent biological VSV vectors [96]. Female *S. vittatum* that fed on virus-rich lesions of VSV-NJ-infected livestock were able to transmit the virus to healthy animals, which subsequently developed clinical disease followed by seroconversion [93]. Also, *S. vittatum* flies transmitted the virus to domestic swine (*Sus scrofa*) immediately after interrupted feeding on a vesicular lesion of an infected host, suggesting that mechanical transmission of VSV-NJ to livestock by black flies is feasible [66]. For intrathoracically inoculated *S. vittatum*, the EIP for VSV-NJ is reported to be 3 days [93], and for orally infected *S. notatum* it is 6 days [94].

In addition, infected black flies have been shown to be capable of transmission to other black flies when feeding nearby on the same non-viremic host or when feeding on sites where infected flies had previously fed [79,97]. After non-infected black flies were allowed to co-feed on an animal adjacent to VSV-NJ infected black flies, a relatively high percentage (26%) became infected, even in cases in which viremia was not subsequently detected in the vertebrate host [79]. These results indicated the acquisition of the virus by co-feeding, regardless of the infection status of the host [79]. Because non-infected and infected vectors often feed on the same host in nature and feed in the same areas on the host, these results have major significance in the maintenance and transmission of VSV in enzootic and epizootic regions and give an insight into how VSV could be maintained when the susceptible hosts produce minimal to no viremia [79].

Studies of VSV transmission by black flies also have helped to elucidate the correlation between the clinical disease course in the vertebrate host and the site of blood feeding by infected flies [56]. Transmission of VSV-NJ by *Simulium* feeding resulted in clinical disease only when feeding sites were those where lesions are usually observed (snout, mouth, and coronary band) [14,56,93]. Bites of VSV-infected black flies at other sites, such as the abdomen or neck, consistently resulted in seroconversion in the absence of lesion formation [14,93]. Moreover, no viremia was detected in black flies after biting the skin of the neck or flank of infected animals [56]. Because these pool-feeding flies naturally prefer feeding on hairless areas, such as the lips, muzzles, and coronary bands, this may suggest that vector transmission of VSV is more important for the development and severity of clinical disease during outbreaks than fomite or direct animal-to-animal transmission [98].

In the western United States, many black fly species are common pests of VSV-susceptible livestock. Moreover, the pattern of a one-year outbreak, or the first year of a two-year outbreak, follows the rivers [7,46,61,62], which are distinct ecological features used as livestock grazing areas and known breeding sites for black flies [7]. The combined results of these field observations and laboratory transmission studies have led to incriminating black flies as important vectors of VSV [9,14,73]. Additionally, the great dispersal distance reach for black fly species (from 225 km to 500 km from their natal habitat) [98] could provide a possible explanation for the spread of VSV out of Central American and Mexico into the U.S.

#### 4.2.4. Biting Midges (Diptera: Ceratopogonidae)

*Culicoides* midges are important agricultural pests and arbovirus vectors, and their role in arbovirus transmission is well-known worldwide. In the U.S., *Culicoides sonorensis* is one of the most common midge species associated with livestock agriculture and has a broad distribution range across the country [99]. Among all the midge species, *C. sonorensis* is of greatest agricultural importance as vectors of several emerging and re-emerging arboviruses, including bluetongue virus and epizootic hemorrhagic disease virus [82]. *Culicoides sonorensis* is able to opportunistically obtain blood meals by pool-feeding on a wide variety of hosts ranging from wildlife to humans [100]. Overall, the importance of *C. sonorensis* as a transmission vector of VSV has been well established [15,16,101,102], and the New Jersey serotype has been isolated from biting midges during epizootics in Colorado and Utah [67,82].

*Culicoides sonorensis* was shown to be susceptible to VSV-NJ infection per os with virus detected in midge tissues throughout the insect, including salivary glands and eggs, for up to 13 days after feeding on an infectious meal [15,16]. The relatively high virus titers in midges suggested significant levels of viral replication and led to incriminating this species as a potential biological vector of VSV [16]. Subsequent studies showed VSV-NJ was able to infect and escape the midgut and salivary gland barriers [15], and midges were able to transmit VSV to guinea pigs [101] and cattle [102]. Moreover, the bite of a single infected midge was able to elicit seroconversion in a guinea pig [101]. Similar to studies with black flies, the phenomena of seroconversion without clinical signs was detected when *Culicoides* bites occurred in sites different from where the lesions are usually observed [101].

Midge species other than *C. sonorensis* have been poorly studied and could be important to the epidemiology of VS. In addition to *C. sonorensis*, *Culicoides stellifer* and other *Culicoides* members of the subgenus, *Selfia*, were positive for VSV in an outbreak in Colorado and Utah in 1982–1983 [82]. *Culicoides stellifer* is a widespread and abundant species that can be found in multiple types of habitats [103], and also species of the subgenus *Selfia* can reach large population numbers [82]. Furthermore, species of *Selfia* are found throughout the southwestern and plains states of Texas, New Mexico, Arizona, Nevada, Utah, Colorado, and Wyoming [104] in congruence with outbreak distributions of VSV. The larval habitat for species of this subgenus is typically small streams [104] and could allow these midges to facilitate dispersal of VSV outwards from large rivers in multiyear outbreaks.

VSV-NJ replication and tissue distribution in *C. sonorensis* are well described, providing insight into the temporal-spatial fate of virus in orally infected midges [15]. After midges fed on a virus-spiked meal, viral replication was detected in tissues of the alimentary canal according to a pattern similar to the route of digestion and absorption. The circulation of virus in the hemolymph by day 3 coincided with infection of the dermis, fat bodies, salivary glands, eyes, cerebral and sub-thoracic ganglia, and ovaries. The short 3-day EIP, along with the disseminated and non-cytolytic infection, is consistent with patterns in an efficient biological vector, particularly when the virus is replicated throughout the insect, passing both midgut and salivary gland infection barriers and reaching transmission-related organs. VSV was shown by in situ detection of mRNA to be replicating in the ovarian epithelium and within the developing oocytes, suggesting that transovarial transmission might be possible [15]. If TOT occurs in midges, then it is likely at a very low rate, or outbreaks would occur annually or at least more often than in 10-year intervals. At most, it may be important in perpetuating overwintering genotypes of VSV, which have been genetically characterized from field isolates during the second year (2014–2015) or third year (2004–2006) of multiple year outbreaks [64]. However, in the absence of infected animal hosts, viruses die out and outbreaks stop until the next successful incursion from Mexico [2].

Similar to what has been proposed in mosquitoes, midge saliva/feeding seems to facilitate VSV transmission and possibly exacerbates pathogenesis. Previous studies have shown that virus transmission to cattle is much more effective when delivered by *C. sonorensis* midges than by needle inoculation [105]. Recent investigations of the biting midge salivary proteome have identified several immunomodulatory proteins [106] and shown a dramatic effect of midge saliva on innate mammalian immune responses post blood-feeding [106,107,108]. Recently, orbiviruses dissemination has been shown to be highly favored when delivered during midge feeding [109]. Vector saliva may be promoting infection through co-feeding processes by impacting the immune response at the local site of feeding [105,110]. Theories have been proposed regarding the possible role of *Culicoides* saliva in the persistence of VSV in the skin or the act of feeding and saliva deposition causing the migration of virus to the skin, but these have yet to be demonstrated [105].

Although vector competence can be estimated in the laboratory, understanding the factors influencing vectorial capacity in the field is difficult and is further complicated by the fact different ecologies influence genetics, physiological traits, and interactions between vector populations and infected hosts [111,112]. Many of the important *Culicoides*-borne viral diseases are strongly linked to environmental factors influenced by climate and weather [112,113]. In particular, the vectorial capacity of *Culicoides* midges is highly impacted by climatic factors (e.g., temperature, precipitation, relative humidity, light intensity, and wind speed) that influence seasonality, fecundity, longevity, distribution, and abundance [114,115]. Therefore, the vectorial capacity of midges for VSV involves a complex interplay between biotic and abiotic factors, and between the insects and the genetic strains of the virus. The dynamics of the interactions of *Culicoides*-VSV will likely vary depending on the different populations and ecologies. For example, the highly competent *C. sonorensis* may be inadequate to spread the virus if the frequency of host contact is low or if certain climatic conditions, such as low temperatures, resulted in a longer EIP needed for VSV transmission. Conversely, a population in ideal temperatures for a shorter EIP, frequent feeding on nearby infected hosts, and high population densities can easily sustain and expand an outbreak. Large population numbers and swarm dispersal by flying or with the help of wind currents [113,115,116] would facilitate the spread of VS, especially into dryer geographic areas away from running water and the black-fly habitat, as is often seen during the second year of a multiple year outbreak [64,73].

## 5. Concluding Remarks

The epidemiology of VSV has a wide variety of variables to consider, including the broad potential vertebrate host range, multiple routes of transmission, variation in clinical outcome in the mammalian host following the site of infection, and diversity of suspected and potential vector species. Additionally, different serotypes and strains have shown independent outbreak patterns, and the vector specificity of the serotypes and strains or if vectors are specific with the geographic dynamics of each outbreak is unclear [117]. The disparity between genetic mutation rates of VSV in laboratories versus outbreaks in natural habitats requires elucidation to better understand the capacity of vesicular stomatitis outbreak serotypes to establish and maintain quasispecies populations in nature.

**Figure 1:**
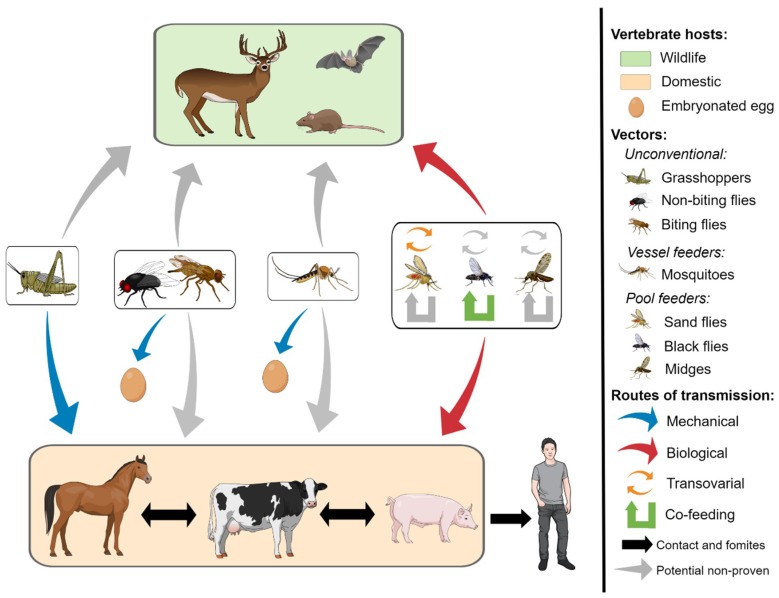
Transmission networks for vesicular stomatitis virus. The arrows represent viral flow between the vectors and hosts involved in known (colored) and proposed (gray) virus transmission cycles. Non-biting flies include houseflies, eye gnats, and anthomyiid flies. Biting flies include horseflies, deer flies, and stable flies. Biological transmission comprises a competent vector becoming infected with vesicular stomatitis virus (VSV) by feeding on blood or feeding on vesicular lesions, amplifying the virus, and transmitting it during subsequent blood-feeding. Mechanical transmission can occur through viral shedding from the alimentary canal, viral-contaminated mouthparts or feet, or by regurgitation of virus from gut contents. Experimentally biting flies and mosquitoes have been shown to transmit VSV to embryonated eggs. Non-conventional routes of transmission include transovarial transmission, co-feeding transmission, or by animals ingesting infected grasshoppers. As described in this review, the ecological characteristics of epizootic sites are key for virus transmission dynamics and some of the elements pictured may not always be present.

In the western U.S., VSV epizootics are sporadic, but consistently correlate with vector seasons [8]. Once in a herd, VS spreads rapidly between animals by direct contact [1,10]. Even though the details of the transmission cycle (Figure 1) are not completely understood, VSV has been isolated from multiple arthropods collected in enzootic areas and during epizootics, demonstrating a biological association in space and time of the suspected vector species with the occurrence of clinical infection in the host [60,67]. Accumulating evidence supports the argument that insect vectors have an important role in the initial introduction of VSV into animal herds and contribute to the epizootic transmission [1,2,7,46,69]. The most implicated primary vector species, black flies and *Culicoides* midges, are known agricultural pests in high abundance at the time when outbreaks occur. Understanding the temporal-spatial distribution and transmission rates of suspected vector species are key to understanding VSV epidemiology.

VSV can infect a wide range of insect species. This promiscuity makes differentiation of inadvertent infections of multiple pest species with transmission-competent vectors difficult. Thus, uncertainty remains regarding if all hematophagous species implicated by host seroconversion after bite-exposure serve as biological vectors of VSV. Additionally, in some cases, experimental designs used to test biological transmission do not constitute relevant routes of insect infection and therefore do not rule out a mechanical transmission-infection route. Differences between naturally fed versus intrathoracically injected insects, in terms of viral replication levels and tissue tropism, has been shown in black flies [96] and biting midges [15], and clearly indicate that experimental oral/ingestion infection is necessary to show VSV vector competence.

Regarding the current evidence for transmission, increasing whole-body VSV titers over time in an insect does not guarantee transmission by a competent vector [118]. Detection of the virus in biological and mechanical transmission-relevant organs, such as salivary glands, eggs, ovarial sheaths, spermatheca, and rectal ampillae, by use of immunohistochemistry is invaluable for indicating specific transmission routes in situ [118]. Indications such as these, along with nucleic acid and live virus detection in salivary glands or in secreted saliva, are necessarily followed by bite transmission studies. Moreover, to reveal generation to generation transmission, detection of the virus in eggs and ovarial tissues is necessary, with confirmation by transovarial transmission studies. Several methods of mechanical transmission by insect species may occur through viral shedding from the alimentary canal, viral-contaminated mouthparts or feet, or regurgitation of the virus in gut contents as shown with bacteria in many filth fly species [119]. As with the bite and transovarial transmission scenarios, quantifiable, reproducible demonstration of live virus transmission by any of these routes is required to advance these beyond speculation.

The complex interrelationships that exist between VSV and some implicated vector species do not strictly align with the requirements of traditional mechanical or biological transmission dogma. Understanding these non-conventional routes of VSV transmission might help explain environmental maintenance mechanisms during inter-epidemic periods and times of adverse conditions for horizontal transmission through biting Diptera or animal-to-animal direct contact. One proposed non-conventional route includes mechanical transmission by VSV-amplifying herbivorous grasshoppers, which can become infected by eating pasture plants contaminated by the saliva of infected grazers, can transmit virus to cattle, which are reported to eat significant numbers in pasturelands and in baled grass/hay, and can migrate hundreds of miles in a season, possibly carrying VSV to pastured herds outside the geographic range of the main vector species [71,72]. Another non-conventional route of VSV transmission occurs during co-feeding and involves infected and uninfected vectors without an infected vertebrate host. As shown in black flies [3,79], this non-systemic co-feeding mechanism may provide an explanation for transmission within insect populations in the absence of clinically infected animals. Transmission rates in the non-systemic pathway are more dependent on time, space, and insect feeding behavior, and might allow for a more rapid increase in viral prevalence in pool-feeders early in the development of a new outbreak. Since non-systemic transmission by co-feeding is particularly important in pool and swarm feeders [3], it is important to examine this phenomenon in additional pool feeding species. Finally, combining direct contact transmission within herds, indirect contact with virally contaminated fomites, horizontal vectored transmission (both mechanical and biological), along with possible vertical transmission (transovarial), as shown in sandflies, increases the range of ecological, temporal, and spatial conditions in which VSV can persist and spread. Any pursuit of a coherent, ecologically-based model to predict VSV outbreaks must consider these multiple vector groups and transmission mechanisms.
